# The mediating role of coping strategies on job satisfaction and burnout and the moderating role of neurotic personality among physicians in Gansu Province’s infectious disease sentinel medical institutions

**DOI:** 10.3389/fpubh.2025.1692851

**Published:** 2025-11-13

**Authors:** Zhiguo Li, Yifei Li, Jinyu Wang, Sheng Li

**Affiliations:** 1 School of Public Health, Gansu University of Chinese Medicine, Lanzhou, China; 2 The First Clinical Medical College, Lanzhou University, Lanzhou, China; 3 School of Public Health, Lanzhou University, Lanzhou, China; 4 The No.2 People’s Hospital of Lanzhou, Lanzhou, China

**Keywords:** physicians, neuroticism, coping styles, burnout, job satisfaction

## Abstract

**Objective:**

This study aimed to investigate the relationship between neuroticism, coping styles, job satisfaction and burnout among physicians in Gansu Province’s infectious disease sentinel medical institutions.

**Methods:**

A cross-sectional survey was conducted using the Minnesota Satisfaction Scale short-form scale, Coping Style Scale, Burnout Scale, and Chinese Big Five Personality Questionnaire short-form scale on 8,071 physicians in all infectious disease sentinel medical institutions in Gansu Province.

**Results:**

There was a correlation between job satisfaction and burnout, coping styles, and neuroticism among doctors in Gansu Province infectious disease designated medical institutions (*p* < 0.05). Coping style mediated the relationship between job satisfaction and burnout. Neuroticism moderated the relationship between job satisfaction and burnout (*t* = −3.231, *p* < 0.05); neuroticism moderated the relationship between job satisfaction and positive coping (*t* = −10.927, *p* < 0.05); neuroticism moderated the relationship between positive coping and burnout (*t* = 4.097, *p* < 0.05); neurotic personality moderated the relationship between negative coping and burnout (*t* = −2.710, *p* < 0.01).

**Conclusion:**

Job satisfaction influences burnout among physicians in an infectious disease sentinel care facility through the mediating role of coping styles and the moderating role of neurotic personality.

## 1 Introduction

In the context of frequent global public health events, physicians, as the core force of epidemic prevention and control, are chronically exposed to high-intensity and high-risk work pressure. Among them, the occupational exposure risk of infectious disease physicians is significantly higher than that of healthcare workers in other departments because they need to be continuously exposed to pathogens (1, 2). Studies have shown that medical staff generally face a significant increase in the risk of burnout, anxiety, and depression during outbreaks (3). Burnout is manifested as emotional exhaustion, depersonalization and reduced sense of personal accomplishment (4), which not only harms the individual health of doctors, but also may lead to a decline in the quality of medical services and even brain drain (5). As the COVID-19 pandemic has demonstrated, physician burnout has become a global concern that threatens the stability and sustainability of healthcare systems. At the same time, job satisfaction, as an important indicator of occupational mental health, directly affects doctors’ work engagement and willingness to stay in their jobs (6). The decline in job satisfaction not only weakens enthusiasm but also intensifies emotional exhaustion and professional burnout, thus forming a vicious cycle. In this context, the role of coping strategies is particularly critical. Coping strategies refer to the cognitive and behavioral regulation adopted by individuals in the face of stress, which can be divided into positive coping (e.g., problem solving, seeking support) and negative coping (e.g., avoidance, self-blame). Studies have shown that positive coping strategies are effective in relieving occupational stress in healthcare workers, while negative coping may exacerbate burnout (7). However, most of the existing studies have focused on the direct effects of coping strategies, and their mediating role between occupational stress and psychological outcomes has been under-explored. In addition, individual difference factors (e.g., personality traits) may moderate this process. Neuroticism, one of the Big Five personality dimensions characterized by emotional instability and anxiety, may amplify the perception of stress and reduce the effectiveness of coping strategies (8). It has been shown that in stressful situations, highly neurotic individuals are more likely to transform positive coping into overdependence or emotional catharsis rather than effective problem solving, which reduces the protective nature of positive coping and leads to burnout (9). Conversely, individuals with lower levels of neuroticism may benefit more from positive coping and maintain higher levels of job satisfaction even in challenging work environments.

Given these findings, it is increasingly recognized that the relationship between job satisfaction and burnout cannot be understood solely as a direct linear process. Instead, it involves a complex interplay of coping strategies and personality traits that shape individual responses to occupational stress. Exploring these mechanisms among infectious disease physicians is particularly important, as this group plays an irreplaceable role in epidemic prevention yet faces chronic high-risk exposure and limited organizational support. A deeper understanding of how coping styles mediate and neuroticism moderates the relationship between job satisfaction and burnout will provide valuable insights for designing tailored interventions that enhance psychological resilience and organizational well-being among frontline healthcare workers.

## 2 Methodology

### 2.1 Sample

This study was a cross-sectional study. The sample covered clinicians engaged in infectious disease prevention and treatment and related specialties in all infectious disease sentinel diagnostic and treatment institutions in Gansu Province, and the online survey was conducted. The online questionnaire survey was conducted between March 27, 2024 and April 20, 2024, and 8,129 questionnaires were distributed, with 8,071 valid data retained after excluding invalid responses, resulting in an effective recovery rate of 99.28%. The inclusion criteria were set as: physicians with one year or more of clinical work experience in infectious diseases; the exclusion categories included: (1) medical personnel in the refresher training stage; (2) enrolled personnel on consecutive leave of absence for more than three months; (3) interns and medical personnel undergoing special refresher training outside. The study protocol has been approved by the Medical Ethics Review Committee of Lanzhou Pulmonary Hospital (Approval No. 2024111901). Before the survey was implemented, the team members communicated and coordinated with the relevant hospital departments to obtain support, and then the leaders in charge took the lead in mobilizing the medical workers to cooperate with them in filling out the questionnaires. In the introductory part of the questionnaire, the purpose of the survey, its content and the requirements for filling it out were explained in detail, emphasizing anonymity and the non-disclosure of any personal privacy information to obtain the trust and informed consent of the survey respondents.

### 2.2 Measurement

#### 2.2.1 Demographic characteristics scale

Contains: socio-demographic characteristics: gender, age, and title basics.

#### 2.2.2 Minnesota satisfaction scale short-form scale(MSQ)

Developed by Weiss et al. (10). Job satisfaction is usually considered in terms of both intrinsic factors (factors that promote job satisfaction, such as opportunities for promotion and growth, recognition, responsibility, and achievement) and extrinsic factors (factors that impede job satisfaction, such as supervision, pay, policies, working conditions, interpersonal relationships, and safety). Thus, in the MSQ, the questionnaire consisted of 20 questions, items 5, 6, 12, 13, 14 and 19 assessed extrinsic satisfaction indicators, items 1–4, 7–11, 15, 16 and 20 assessed intrinsic satisfaction indicators while the other two items 17, 18 were used to determine general job satisfaction. A likert 5-point scale was used: very dissatisfied, dissatisfied, uncertain, satisfied, and very satisfied, corresponding to a score of 1 to 5, with the closer to 1 indicating more dissatisfaction, the closer to 5 indicating more satisfaction, and higher scores indicating better occupational satisfaction. The Cronbach’s alpha coefficient for this scale in this study was 0.965.

#### 2.2.3 Simplified coping style questionnaire(SCSQ)

Based on the revised SCSQ by Xie Yaning (11), it contains two dimensions of positive coping (questions 1–12) and negative coping (questions 13–20). A four-point Likert scale was used: 0 = not used, 1 = occasionally used, 2 = sometimes used, and 3 = often used, with higher mean scores on the positive coping dimension indicating that the individual tends to adopt positive coping styles, and vice versa for more negative coping styles. The Cronbach’s alpha coefficient for the scale of this study was 0.831.

#### 2.2.4 Maslach burnout inventor-human service survey(MBI-HSS)

In this study, the Chinese version of the Burnout Scale, which was Chineseized by Huang (12), was used to understand the burnout status of the study participants. The entire scale consists of 22 entries, including 3 dimensions: emotional exhaustion, dehumanization, and low personal accomplishment. The Emotional Exhaustion subscale consists of 9 questions, Dehumanization consists of 5 questions, and Low Personal Achievement consists of 8 questions. It was self-rated on a 7-point Likert scale with 0 = “never,” 1 = “about a few times a year,” 2 = “about once a month,” 3 = “about a few times a month,” and 3 = “about a few times a month.” “about a couple times a month”, 4 = “about once a week”, 5 = “about a couple times a week”, and 6 = “every day”. The higher the score on the emotional exhaustion and depersonalization dimensions, the more severe the burnout, and vice versa, the less severe the burnout; the lower the score on the personal accomplishment dimension, the higher the burnout, and vice versa, the lower the burnout. The Cronbach’s alpha coefficient for this scale in this study was 0.895.

#### 2.2.5 The Chinese big five personality questionnaire short version (CBF-PI-B)

The table has a total of 40 entries, of which the neurotic (N) personality traits include entries 1, 6, 11, 16, 21, 26, 31, and 36 entries, which are scored on a six-point Likert scale (1 = not at all, 2 = mostly not, 3 = somewhat not, 4 = somewhat, 5 = mostly, and 6 = completely) (13). Thirty-six of the questions were reverse scoring questions requiring reverse conversion scoring treatment. The Cronbach’s alpha coefficient for the neuroticism dimension in this study amounted to 0.770.

### 2.3 Data collection and quality control methods

The data was collected electronically through “Wenjuanxing” (a popular online survey platform in China). During these meetings, the research objectives, evaluation criteria, academic relevance, and operational procedures became clearer. Special attention was paid to maintaining the confidentiality and anonymity of the participants while strictly following the established inclusion/exclusion criteria. After obtaining informed consent, the participants completed the questionnaire anonymously by email. Participants could only submit one questionnaire per device and IP address to avoid duplicate responses. To ensure comprehensiveness, every question must be answered. Each questionnaire is strictly reviewed after collection. To improve data quality, those who gave predictable responses (such as alternating or linear responses) or completed the task in less than three minutes will be disqualified.

### 2.4 Statistical methods

The data of this study were analyzed using SPSS 26.0 statistical software. The data were presented as median (interquartile spacing) [M(P25, P75)] after confirming that the data were non-normally distributed by normality test. General demographic characteristics were analyzed using descriptive statistics, mediating effects were analyzed using correlation analysis, and regression analyses were performed using the SPSS macro program Process 4.1 plug-in *α* = 0.05.

### 2.5 Model assumptions

**Figure 1:**
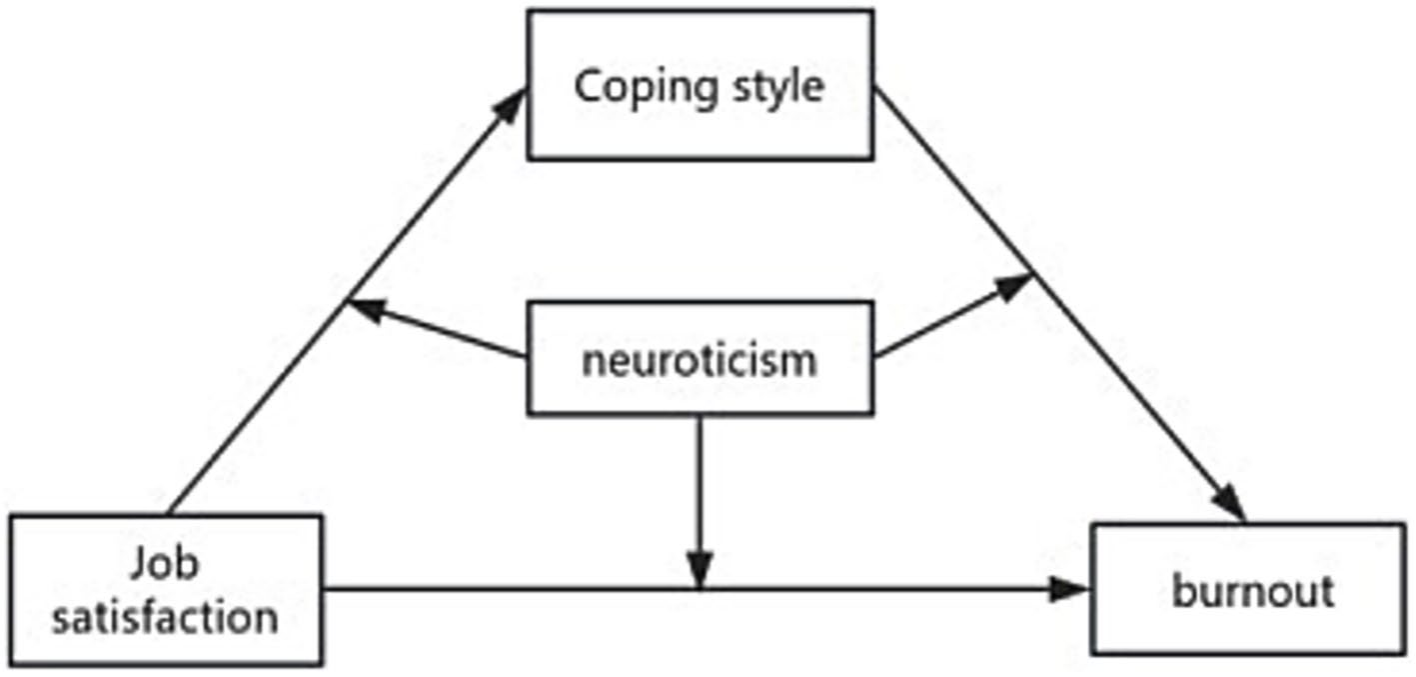
Diagram of model assumptions.

Due to issues related to job burnout and job satisfaction, not only do they affect an individual’s health and career development, but they also directly relate to the resilience of the public health system. Therefore, constructing a comprehensive model of mediating and moderating variables (Figure 1), which takes job satisfaction as the independent variable (X), job burnout as the dependent variable (Y), coping styles as the mediating variables (M1 and M2), and neurotic personality as the moderating variable (W), is helpful for a more comprehensive revelation of the dynamic mechanism of the professional psychology of infectious disease doctors.

## 3 Results

### 3.1 Demographic characteristics

A total of 8,071 physicians from designated medical institutions for infectious diseases were selected for the study. Among them, 4,455 were female and 3,616 were male; 2,608 doctors were ≤30 years old, 2,885 were 31–40 years old, 1,685 were 41–50 years old, and 893 were >50 years old. The titles of the doctors were 4,442 doctors at junior level and below, 2,125 doctors at intermediate level, 1,206 doctors at deputy senior level and 298 doctors at full senior level. See Table 1.

**Table 1:** Demographic characterization of infectious disease sentinel care facilities (*n* = 8,071).

Variable	Frequency	Percent
Gender		
Male	3,616	44.80
Female	4,455	55.20
Age		
≤30	2,608	32.31
31 ~ 40	2,885	35.75
41 ~ 50	1,685	20.88
>50	893	11.06
Title		
Junior and below	4,442	55.04
Middle level	2,125	26.33
Deputy high level	1,206	14.94
High level	298	3.69

### 3.2 Correlation analysis

Bivariate analysis was used to analyze the relationship between job satisfaction and coping style and burnout. The results of correlation analysis showed that job satisfaction was negatively correlated with burnout (*r* = − 0.519, *p* < 0.01), positively correlated with positive coping (*r* = 0.462, *p* < 0.01), and negatively correlated with negative coping (*r* = − 0.191, *p* < 0.01). In addition, positive coping was negatively correlated with burnout (*r* = − 0.382, *p* < 0.01) and negative coping was positively correlated with burnout (*r* = 0.248, *p* < 0.01) (Table 2).

**Table 2:** Correlation analysis (*n* = 8.071).

Variable	Job satisfaction	Neuroticism	Positive coping	Negative coping	Burnout
Job satisfaction	1.000				
Neuroticism	−0.245**	1.000			
Positive coping	0.462**	−0.131**	1.000		
Negative coping	−0.191**	0.177**	−0.015	1.000	
Burnout	−0.519**	0.489**	−0.382**	0.248**	1.000

**P<0.01.

### 3.3 The mediating role of coping styles

This study adopts the method proposed by Zhonglin and Baojuan (14) to test the mediation model in two steps. The mediating role of the model was tested through Bootstrap technique by choosing job satisfaction as the X variable, burnout as the Y variable, positive coping styles and negative coping styles as the M1 and M2 variables, and gender, age, and job title as the control variables. The results showed that job satisfaction had a significant effect on burnout, positive coping, negative coping, and positive coping (*p* < 0.01), and that this effect did not diminish even after adding the mediating variable of coping styles. In addition, none of the 95% confidence intervals for bootstrap contained 0, further demonstrating the fit of the mediation model. The percentage of direct effects was 77.12%, while the percentage of mediating effects for positive and negative coping was 16.83 and 6.05%, respectively. See Tables 3, 4.

**Table 3:** Mediator variable test: response style (*n* = 8,071).

Variable	Burnout		Burnout		Positive coping		Negative coping	
	*t*	*P*	*t*	*P*	*t*	*P*	*t*	*P*
Gender	−5.028	<0.001	−6.579	<0.001	1.655	0.097	−7.218	<0.001
Age	−9.235	<0.001	−9.209	<0.001	1.772	0.076	0.271	0.786
Title	1.502	0.133	0.342	0.732	4.534	<0.001	−1.139	0.254
Job satisfaction	−37.691	<0.001	−54.351	<0.001	46.808	<0.001	−17.006	<0.001
Positive coping	−18.199	<0.001						
Negative coping	17.791	<0.001						
*R* ^2^	0.334		0.285		0.221		0.043	
*F*	674.143		802.930		570.568		90.257	

**Table 4:** Tests for total, direct and mediating effects variables (*n* = 8,071).

Effect type	*b*	SE	*P*	Bootstrap 95%CI
Indirect effect: job satisfaction → Positive coping → burnout	−0.117	-	-	(−0.131 ~ −0.104)
Indirect effect: job satisfaction → Negative coping → burnout	−0.042	-	-	(−0.049 ~ −0.035)
Direct effect: job satisfaction → burnout	−0.536	0.014	<0.001	(−0.563 ~ −0.508)
Total effect: job satisfaction → burnout	−0.695	0.013	<0.001	(−0.720 ~ −0.670)

### 3.4 The moderating role of neurotic personality

**Figure 2:**
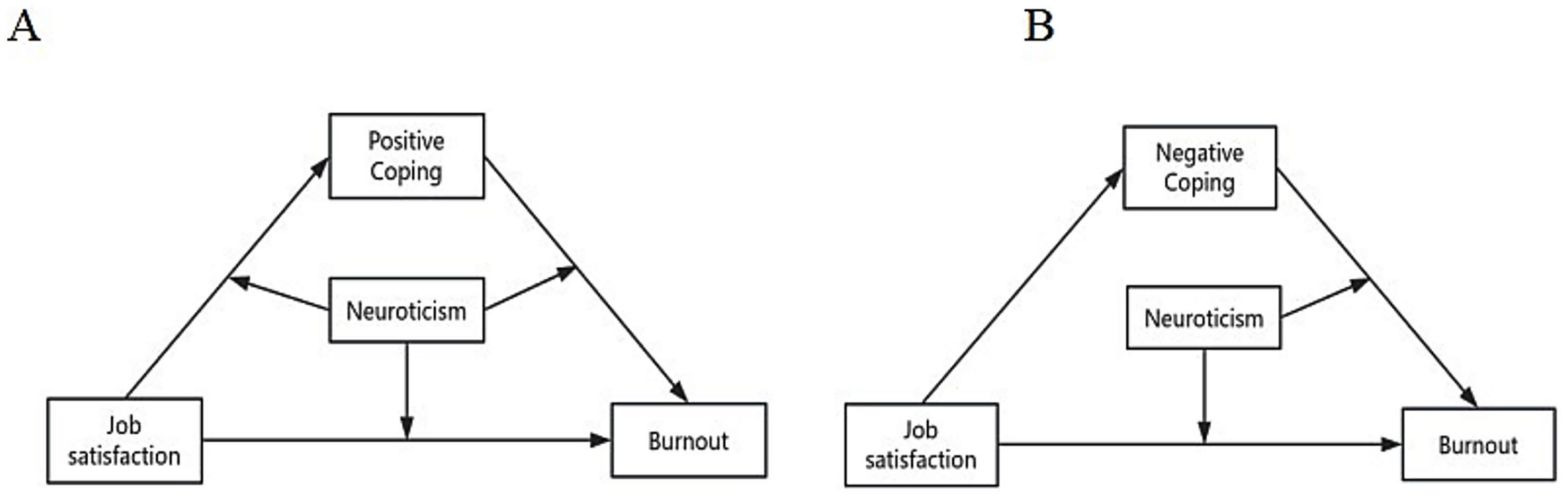
Important pathways regulating neurotic personality in the model.

In order to test a significant mediating model, the second step is to add moderating variables. This study used model 59 to add neurotic personality as a moderating variable, while X, Y, and control variables were held constant from the first step (Figure 2), which showed that neurotic personality moderated the relationship between job satisfaction and burnout (*t* = −3.231, *p* < 0.05); neurotic personality moderated the relationship between job satisfaction and positive coping (*t* = −10.927, *p* < 0.05); neurotic personality moderated the relationship between positive coping and burnout (*t* = 4.097, *p* < 0.05); and neurotic personality moderated the relationship between negative coping and burnout (*t* = −2.710, *p* < 0.05). See Table 5.

**Table 5:** Mediation test with moderation (*n* = 8,071).

Variable	Positive coping			Negative coping			Burnout		
	*b*	Se	*t*	*b*	Se	*t*	*b*	Se	*t*
Gender	0.239	0.146	1.639	−0.668	0.088	−7.612*	−2.385	0.334	−7.135*
Age	0.186	0.104	1.801	0.007	0.062	0.114	−2.565	0.236	−10.855*
Title	0.534	0.118	4.537^*^	−0.059	0.071	−0.831	0.695	0.269	2.585*
Positive coping							−0.473	0.026	−18.516*
Positive Response× Neuroticism							0.013	0.003	4.097*
negative coping							0.566	0.042	13.357*
Negative coping × neuroticism							−0.015	0.006	−2.710*
Job satisfaction	0.220	0.005	43.474*	−0.042	0.003	−13.618*	−0.431	0.013	−32.947*
Job satisfaction×neuroticism	−0.006	0.001	−10.927*	−0.001	0.000	−1.531	−0.005	0.001	−3.231*
Neuroticism	−0.006	0.010	−0.583	0.076	0.006	12.73*	0.979	0.023	42.595*

*P<0.05.

### 3.5 Slope plot of moderating effects

**Figure 3:**
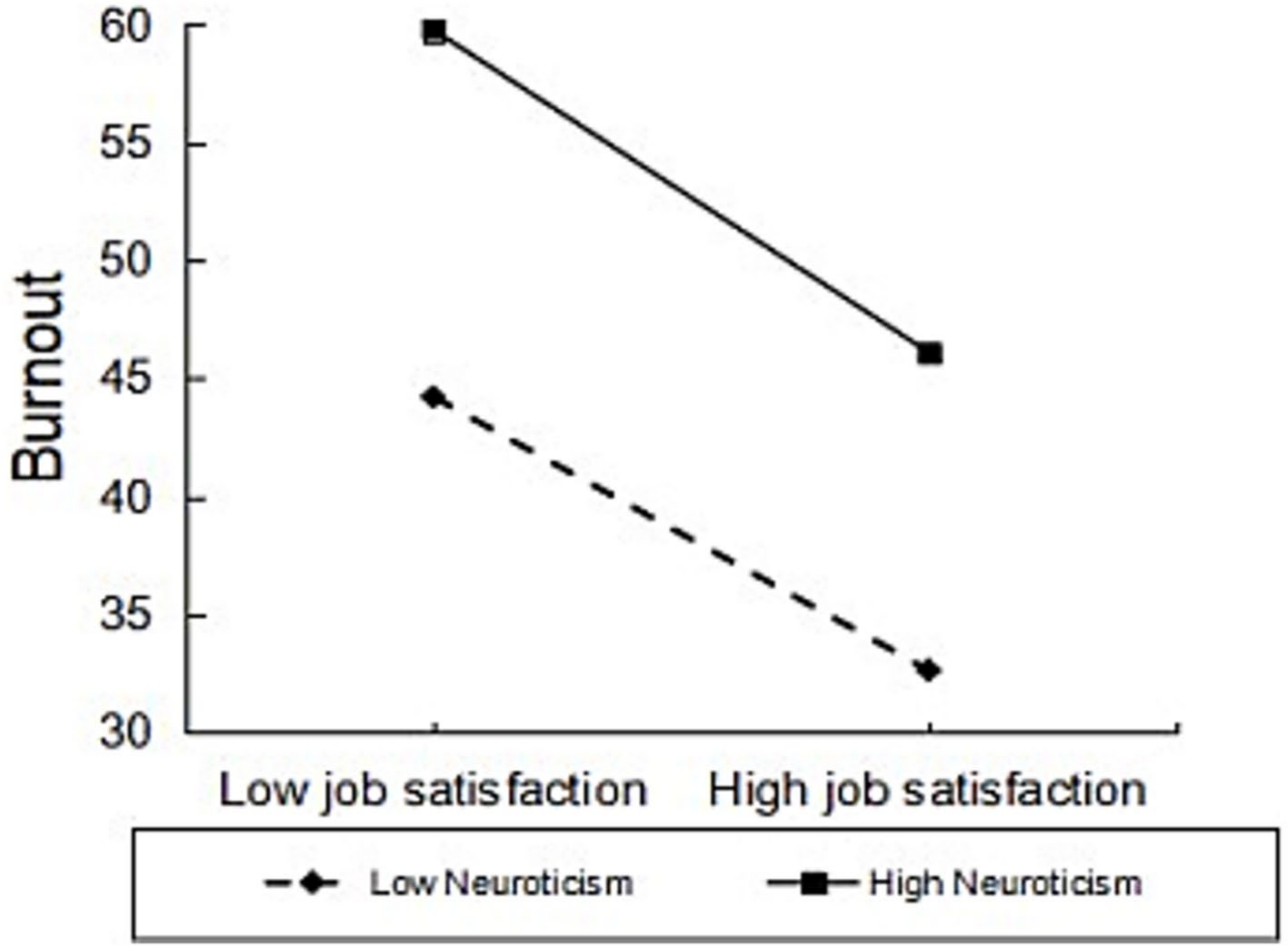
Moderating role of neurotic personality in job satisfaction and burnout.

**Figure 4:**
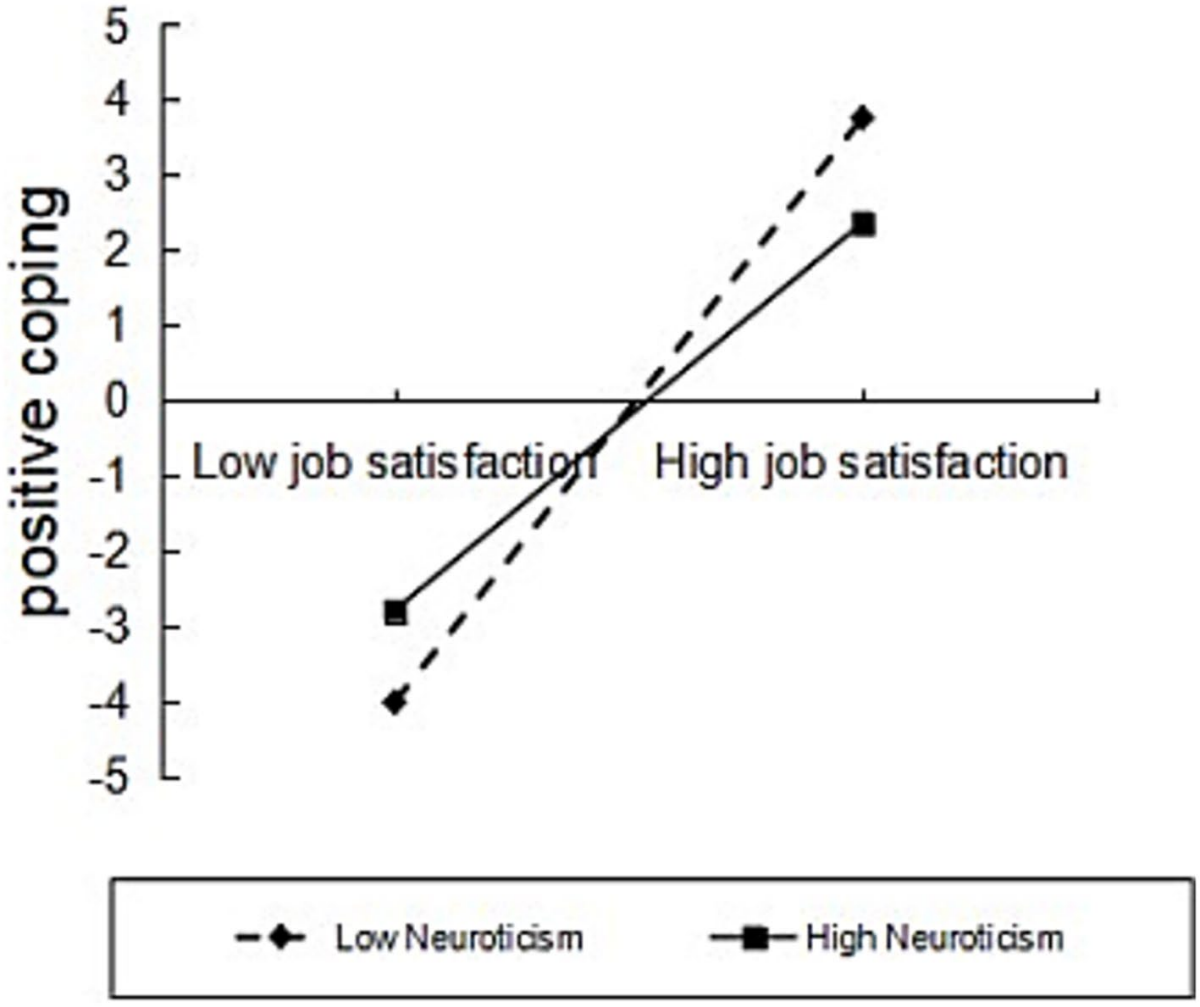
Moderating role of neurotic personality in job satisfaction and positive coping.

**Figure 5:**
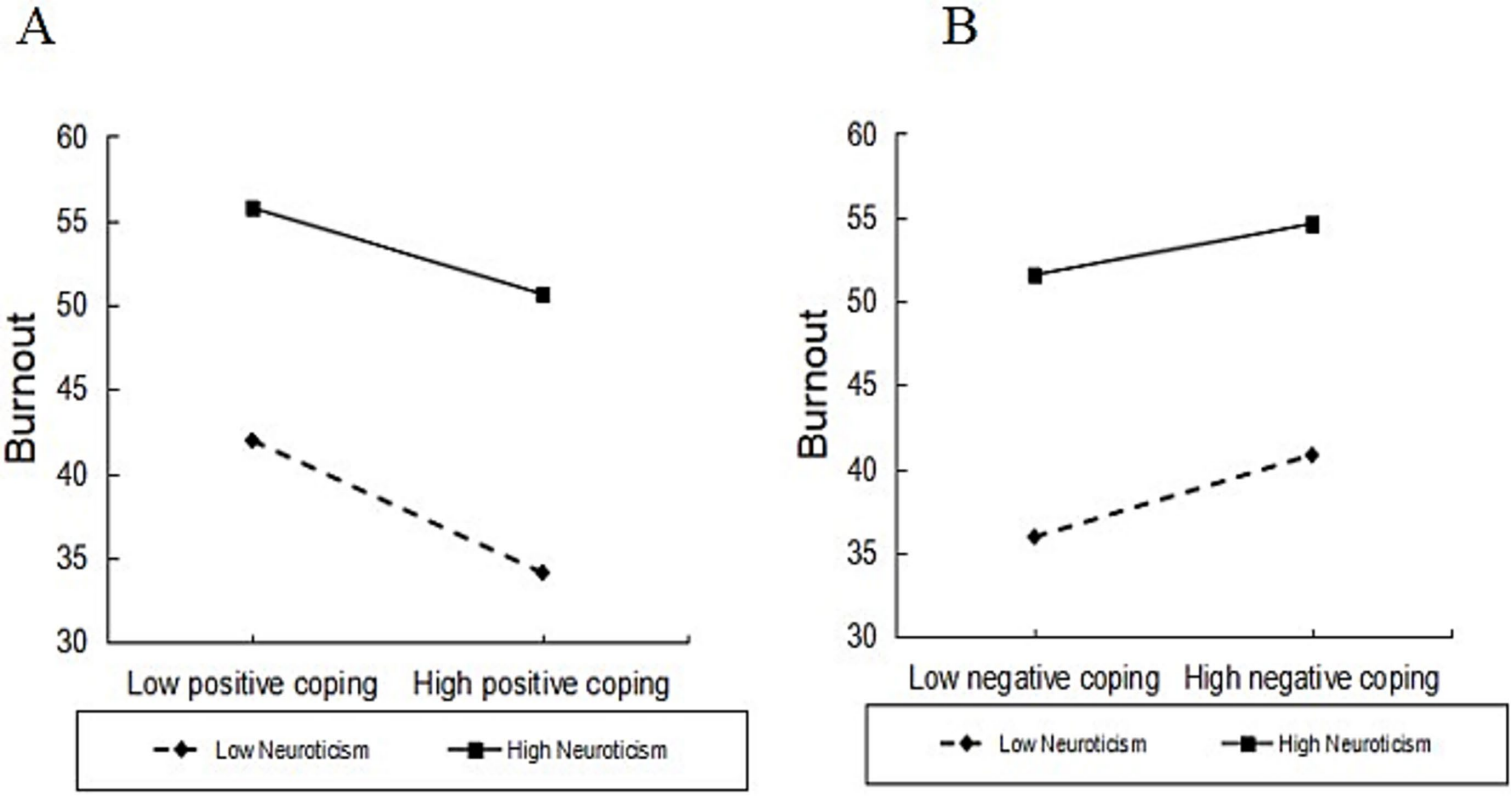
Moderating role of neurotic personality in coping styles and burnout.

Using coping styles as the mediating variable, simple slope plots indicated that burnout was significantly higher among physicians with high neurotic personality than among those with low neurotic personality (Figure 3). Using positive coping styles as the mediating variable, further simple slope plot analyses indicated that both high and low neurotic personality physicians employed positive coping styles increasingly with increasing job satisfaction, and that job satisfaction had a greater predictive effect on positive coping for physicians with low neurotic personality compared to physicians with high neurotic personality (Figure 4); and that burnout was significantly higher for physicians with high neurotic personality physicians had significantly higher burnout than physicians with low neurotic personality (Figure 5A). Using negative coping as a mediating variable, simple slope plots indicated that burnout was significantly higher for physicians with high neurotic personality than for physicians with low neurotic personality (Figure 5B). This means that at the same level of job satisfaction and coping styles, the level of burnout is higher for doctors with high neurotic personality than for doctors with low neurotic personality.

## 4 Discussion

After controlling for confounding variables such as gender, age, and professional title, this study found that job satisfaction, coping styles, and job burnout were significantly correlated. It further confirmed the mediating role of positive/negative coping in the path from job satisfaction to burnout. Additionally, neurotic personality exhibited significant moderating effects on several key relationships in the above path (job satisfaction → burnout, job satisfaction → positive coping, positive/negative coping → burnout).

### 4.1 The mediating role of coping styles

This study found that coping styles play a mediating role between job satisfaction and burnout among physicians in infectious disease sentinel care organizations. Specifically, positive coping styles had a significant negative predictive effect on burnout, while negative coping styles were positively associated with burnout. This echoes the findings of the Kupcewicz and Jóźwik study, where positive coping styles have also been shown to alleviate job stress and reduce burnout levels in a group of nurses (15). In a study of healthcare workers exposed to 2019 coronavirus disease, it was found (16) that healthcare workers are at risk of infection due to direct contact with patients in the treatment of infectious diseases, as well as intensive work and frequent night shifts, leading to physical and mental exhaustion and emotional depletion. Proactively seeking support is one of the most important strategies for physicians to cope with burnout. A systematic review and analysis showed (17) that burned-out physicians scored significantly higher on defensive practices (e.g., avoidance behaviors) than those without burnout, and that physicians who were proactively exposed to psychological interventions showed significant improvements in emotional exhaustion, dehumanization, and overall burnout. Stewart’s coping theory states that social support should be identified as one of the coping resources (18). Among Chinese physicians, social support indirectly reduces negative coping by decreasing negative emotions, thus enhancing job satisfaction (19). This suggests that when physicians feel stressed or burned out, they are more likely to engage in avoidance behaviors, such as avoiding patient contact or reducing work engagement. This behavioral pattern may seem to temporarily alleviate psychological stress in the short term, but in the long run, it will weaken doctors’ professional commitment and self-efficacy, further exacerbating the vicious cycle of occupational burnout. Furthermore, negative coping strategies may also weaken the collaborative atmosphere among teams and the sense of organizational support, making individuals more isolated when facing work challenges (20). Studies have shown that medical staff with strong positive coping abilities can better maintain their mental health levels under high-pressure situations, thereby effectively reducing emotional exhaustion (21).

In terms of the specific percentage of mediating effect, the mediating effect of positive coping is 16.83%, while the mediating effect of negative coping is 6.05%. This indicates that the mediating effect of positive coping between job satisfaction and burnout is relatively more significant. It has been shown (22) that the level of burnout during the epidemic was significantly lower among physicians who used positive coping than among the group who relied on negative coping. This is precisely because positive coping styles are more effective in helping physicians cope with various problems at work, increasing their sense of control and fulfillment at work, and thus alleviating burnout. It can be seen that in the special working environment of infectious disease designated medical institutions, doctors face great work pressure and psychological burden, and positive coping strategies such as actively seeking support and social support can help them better cope with the challenges at work, thus increasing job satisfaction and reducing the risk of burnout. On the contrary, negative coping strategies such as avoidance and complaining may lead to lower job satisfaction and increase the risk of burnout.

### 4.2 The moderating role of neurotic personality

Neurotic personality was shown to have a moderating effect on the relationship between job satisfaction and burnout in this study, as well as on the relationship between job satisfaction and coping styles, between positive coping and burnout, and between negative coping and burnout. Specifically, neurotic personality moderated the relationship between job satisfaction and burnout, with physicians with high neurotic personality having higher levels of burnout when job satisfaction was low, and relatively lower levels of burnout when job satisfaction was high, but still higher than physicians with low neurotic personality. A study of older adults Chinese nurses found (23) that highly neurotic individuals were less able to perceive positive experiences, making it difficult for increased job satisfaction to offset the accumulation of negative emotions. This suggests that physicians with higher neurotic personalities are more sensitive to job stress and are more likely to experience burnout in the presence of lower job satisfaction. According to Huang’s study (24), neurotic doctors are more likely to pay attention to frustrating details of their work (e.g., doctor-patient conflicts) and ignore positive feedback, resulting in a weakening effect of “satisfaction-burnout.” This is consistent with our findings. Neurotic personality weakened the effect of job satisfaction on positive coping.

Neurotic personality also moderated the relationship between job satisfaction and positive coping. The predictive effect of job satisfaction on positive coping was greater for physicians with low neurotic personality. It has been shown (25) that high-neuroticism individuals often choose impulsive or avoidance strategies due to negative problem orientation, whereas low-neuroticism individuals are more adept at integrating new information with routine demands to enhance problem-solving efficiency. This also suggests that physicians with low neurotic personality are better able to maintain a positive mindset and actively seek solutions to problems in the face of work stress, thus better coping with challenges at work. On the other hand, doctors with high neurotic personality have difficulty in coping with problems at work as positively as doctors with low neurotic personality due to their poor emotional stability, even if they have higher job satisfaction. In addition, neurotic personality moderated the relationship between positive coping and burnout, as well as the relationship between negative coping and burnout. This further illustrates the important role that neurotic personality plays in physicians’ coping with job stress and burnout. Doctors with higher neurotic personality have difficulty in effectively alleviating burnout even when they adopt positive coping styles in the face of job stress, and the degree of burnout is further exacerbated when they adopt negative coping styles. According to Wang’s study (9), neuroticism has the highest expected influence on the bridge to burnout among the Big Five personalities, and it is significantly and positively correlated with skepticism of significance and sense of worthiness. Fendel’s study shows (26) that although positive thinking training significantly reduces emotional exhaustion scores of general practitioners, the effect is weaker for highly neurotic doctors. This precisely suggests that physicians with neurotic personalities are more likely to be trapped in a burnout cycle due to negative cognitions, and that their intrinsic negative thought patterns may still undermine the effectiveness of the intervention even when positive actions are taken. This may be due to the fact that physicians with higher neurotic personalities are more prone to negative emotions, such as anxiety and depression, when faced with stress, which can weaken the effects of positive coping styles while exacerbating the effects of negative coping styles on burnout.

This regulatory effect further highlights the central role of personality traits in occupational mental health. Neuroticism, as a stable individual difference variable, not only influences the emotional response patterns of individuals when facing stress, but also profoundly shapes their cognitive evaluations and behavioral choices. Highly neurotic doctors, when confronted with high-pressure situations such as complex doctor-patient relationships, work intensity, and public health events, often exhibit stronger threat perception and negative attribution tendencies, thereby weakening the sustainability and effectiveness of positive coping.

This study has some limitations: the cross-sectional questionnaire did not allow for causal inference, and a longitudinal study could be used in the future to validate causality, thus confirming the lagged effect of the relationship between chronological order and identifying variables.

## 5 Conclusion

Job satisfaction influences physician burnout in infectious disease sentinel care facilities through the mediating role of coping styles and the moderating role of neurotic personality. Positive coping reduces the negative impact of low job satisfaction on burnout, whereas negative coping aggravates emotional exhaustion. Physicians with high neuroticism are more sensitive to stress and less likely to use positive coping, increasing their burnout risk. These findings suggest that enhancing positive coping skills and providing psychological support—especially for individuals with high neuroticism—can effectively improve job satisfaction, reduce burnout, and promote physicians’ mental well-being.

## Data Availability

The raw data supporting the conclusions of this article will be made available by the authors, without undue reservation.
