# Supplementary material for: The mediating role of coping strategies on job satisfaction and burnout and the moderating role of neurotic personality among physicians in Gansu Province’s infectious disease sentinel medical institutions
Source: Front Public Health. 2025 Nov 13;13:1692851. doi: 10.3389/fpubh.2025.1692851 (PMC12657139; doi:10.3389/fpubh.2025.1692851)
Supplement: Supplementary file 1 [file Table_1.docx]

**Internet Consent Form**

Dear Participants:

Greetings! We are conducting an online questionnaire survey aimed at exploring the relationship between the job satisfaction of doctors at designated infectious disease hospitals in Gansu Province, their neurotic personality traits, coping styles, and occupational burnout. Your participation will provide us with valuable information, which will help improve mental health and reduce occupational burnout in the medical work environment.

Before proceeding, please carefully read the following information to ensure your full understanding and voluntary consent.

**I. Purpose and Background of the Study**

This study aims to: Explore the mediating role of doctors' coping strategies in job satisfaction and burnout;Assess the moderating effect of doctors' neurotic personality in this model; Identify actionable strategies to enhance mental health support for healthcare workers.

Your responses will contribute to academic research and potential policy recommendations for hospital management.

**Ⅱ. Questionnaire Content and Form**

This questionnaire is filled out online, and all questions are designed based on the research purpose, aiming to obtain real and objective data. You can complete the questionnaire at any time and place through computers, cell phones, or other terminal devices.

**Ⅲ.Data Confidentiality and Privacy Protection**

We promise to keep your personal information and answers to the questionnaire strictly confidential. Your name, contact information, IP address, and other sensitive information will not be recorded or disclosed.

All data will be used only for the purpose of this study and will be anonymized at the end of the study. We will not use your data for any other purpose or disclose it to third parties.

We will take the necessary technical and administrative measures to ensure the security and integrity of the data and to prevent unauthorized access, alteration, or disclosure of the data.

**IV. Voluntary Participation and Withdrawal**

Participation in this online survey is completely voluntary, and you have the right to choose whether to participate and withdraw at any time.

Your withdrawal will not affect any of your rights and interests, nor will it have a negative impact on this study. You can stop completing the questionnaire at any time without providing any reason or explanation.

**V. Contact and Feedback**

If you have any questions or suggestions about this study, please contact us via the following methods:

Email: 1315718562@qq.com

We will answer your questions and provide necessary assistance as soon as possible.

**VI. Other Notes**

Please fill in the questionnaire according to your real situation and feelings to ensure the accuracy and reliability of the data.

When filling out the questionnaire, please pay attention to the protection of your privacy and information security to avoid the disclosure of sensitive personal information.

If you encounter any problems or difficulties in the process of filling out the questionnaire, please feel free to contact us, and we will provide you with the necessary support and assistance.

**Participant Acknowledgment:**

☑ I have read and understood the content of the above informed consent form.

☑ I agree to participate in this online questionnaire and authorize the researcher to process my data according to the above requirements.

Date:__

(Note: Please check the appropriate box to confirm informed consent and participation.)

Thank you for supporting this important research! Your input will help improve the well-being of healthcare professionals in Lanzhou.

Participants in the survey fill out the questionnaire voluntarily in accordance with the requirements of the aforementioned“informed consent form" and may withdraw at any time during the process of filling out the questionnaire.
